# Minimal and normal-flow general anesthesia in patients undergoing surgery in prone position: ımpact on hemodynamics and regional cerebral oxygenation

**DOI:** 10.1590/acb380523

**Published:** 2023-03-24

**Authors:** Esra Akdaş Tekin, Fethi Gültop, Nihan Altıntepe Başkurt

**Affiliations:** 1 University of Health Sciences Prof. Dr. Cemil Tascioglu City Hospital – Department of Anestesiology and Reanimation – Istanbul, Turkey.

**Keywords:** Hemodynamics, Spectroscopy, Near-Infrared, Prone Position

## Abstract

**Purpose:**

In this study, the aim to assess the combined effects of prone-positioning (PP) and minimal-flow (MF) general anesthesia on regional cerebral oxygenation (RCO) and systemic hemodynamics.

**Methods:**

This is a randomized prospective study aiming to evaluate changes in cerebral oxygenation and hemodynamic parameters in MF systemic anesthesia in patients undergoing surgery in PP. Patients were randomized to MF or normal-flow (NF) anesthesia. In the operating room, pulse rate, mean arterial pressure (MAP), peripheral hemoglobin oxygen saturation (spO_2_), and right- and left-side RCO (assessed by near-infrared spectroscopy, NIRS) were measured perioperatively.

**Results:**

Overall, 46 patients were included (24 in the MF group and 22 in the NF group). The amount of anesthetic gas consumption was significantly lower in the low-flow (LF) group. In both groups, the mean pulse rate showed a decrease after PP. Before induction, RCO was significantly higher both at the right- and left-sides in the LF group compared to the NF group. This difference continued throughout the operation on the left-side and disappeared 10 min after intubation on the right-side. On the left side, mean RCO decreased after PP in both groups.

**Conclusions:**

MF anesthesia in PP did not reduce cerebral oxygenation compared to NF and was safe in terms of systemic hemodynamics and cerebral oxygenation.

## Introduction

The rate of utilization of low-flow (LF) anesthesia has gradually increased during the last decade, owing to some clinical advantages and cost savings. First suggested by Baum and Aitkenhead^1^, the benefits of LF anesthesia include, but are not limited to, the following: better body temperature maintenance, better humidity of the respiratory tract with improved mucociliary clearance and respiratory function, decreased amount of inhaled anesthetic use with attendant lower cost, and less environmental pollution^2^. In minimal-flow (MF) anesthesia, the benefits mentioned associated with LF anesthesia is still available with added economic and environmental benefits^2^. The most feared potential complication is the risk of hypoxia due to a hypoxic gas mixture. However, with recent developments in the design of anesthesia machines and sophisticated monitoring tools measuring individual gas flows and concentrations, LF and MF anesthesia are now considered to be relatively safe modalities of inhaled anesthesia.

Actually, the most vulnerable organ to anesthesia-induced hypoxia is the brain. Thus, monitoring of cerebral oxygenation under various settings of general anesthesia is of paramount importance to prevent inadvertent hypoxia-related complications after surgery. Pulse oximeter saturation measurement may not always reflect the cerebral oxygen saturation. In this regard, methods measuring cerebral oxygenation are of value. Near-infrared spectroscopy (NIRS) measures regional cerebral oxygen saturation^3^. This technique has been used successfully to evaluate potential changes in cerebral perfusion and oxygenation under various types of general anesthesia settings^4^. Moreover, managing oxygenation based on intraoperative cerebral oxygenation data obtained via NIRS has been shown to be associated with better postoperative cognitive performance^5^. Given it is the prime concern during LF and MF anesthesia, cerebral oxygenation has been evaluated via NIRS during LF general anesthesia^6^^,^ 7.

Prone positioning (PP) is required in a number of surgical operations to be able to gain adequate access to the operation site. Moreover, PP is frequently used in the setting of acute respiratory distress syndrome thanks to its mechanistic advantages on gas exchanges in the lungs^8^. However, the effects of PP on cerebral perfusion and oxygenation have not been studied well. In a prospective observational study design, Babakhani and colleagues^9^ investigated the effects of PP on cerebral oxygenation under general anesthesia. The authors reported clinically unimportant reductions in bilateral cerebral oxygenation values when patients were positioned from supine to prone. They concluded PP under general anesthesia did not lead to cerebral oxygen desaturation and can be regarded as a safe technique. Another study also confirmed the findings of the latter study in patients who underwent orthopedic surgery under general anesthesia^10^.

Although the impact of LF and MF anesthesia on cerebral oxygenation has been evaluated under various settings of general anesthesia^7^^,^ 11, to the best of our knowledge, no study has investigated the potential effects of MF anesthesia in patients undergoing prone surgery yet. Thus, the objective is to assess the combined effects of PP and MF general anesthesia on cerebral oxygenation in a prospective randomized comparative study. Cerebral oxygenation was evaluated by NIRS in addition to monitoring of other hemodynamic parameters in patients operated in a prone position.

## Material and Methods

### Design, participants, and setting

This was a randomized prospective comparative study that aimed to evaluate the likely changes in cerebral oxygenation and hemodynamic parameters with the use of minimal fresh gas flow systemic anesthesia in patients undergoing surgery in a PP. The study protocol was approved by the local ethics committee decision no: 1477 (05/11/2019) and the Clinical Trials registration was submitted retrospectively on 18/07/2022 and its ID number was NCT05462327. All patients were informed about the study protocol and signed written informed consent forms before enrolling in the study. All study procedures were performed in full compliance with the principles of the Declaration of Helsinki.

Participants in this study are fully informed of the purpose and nature of the study and which data involves the use of vital and laboratory values before and after surgery. It was explained to the participants that within the scope of the study, no additional examination (except for the examinations required for your disease) and tissue and biopsy material would not be requested. The participants were informed that there would be no change or disruption in their treatment if they refused to participate in the study or gave up later. It has been promised that the personal information of the participants (name and surname, etc.) identifying images or other personal or clinical details that compromise anonymity will not be published anywhere, shared with any institution or organization, and will not be disclosed to third parties.

**Figure 1:**
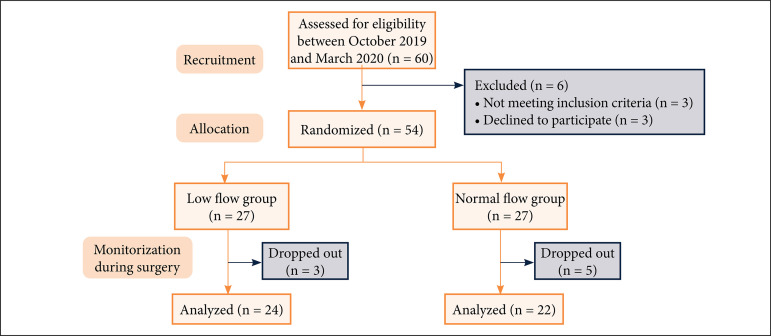
CONSORT flow diagram.

The study was carried out in operating rooms of orthopedics and traumatology and neurological surgery at Okmeydanı Training and Research Hospital, Istanbul, Turkey. Patients were recruited for the study between October 2019 and March 2020 as shown in the CONSORT flow diagram (Fig. 1). Inclusion criteria involved age > 18 years, undergoing surgery in the prone position, having an American Society of Anesthesiologists (ASA) physical status I to III, and willingness to participate in the study. Patients who had one or more of the following features were excluded from the study: having a Glasgow coma scale score ≤ 12, previous history of cranial surgery, advanced cardiovascular and/or pulmonary disease, mental retardation, and neurological disease.

### Anesthesia protocols

Patients were randomly allocated to either minimal fresh gas flow (MF, 0.5 L/min during maintenance) or normal-flow (NF, 2 L/min) general anesthesia. In both groups, participants were first administered with 100% oxygen at a 4 L/min flow rate. Propofol 2–2.5 mg/kg, rocuronium bromide 0.6 mg/kg, and fentanyl 1 mcg/kg IV were used for anesthesia induction. For all general anesthesia procedures, the Drager perseus A500 (Lübeck, Germany) and the Dragersorb were used as anesthesia devices and soda lime, respectively. During induction, all patients were given sevoflurane inhaler anesthesia at a 4 L/min flow rate (FiO_2_ 50%) for 10 min until having a minimal alveolar concentration (MAC) of 0.9. Then, in the MF group, the flow rate was reduced to 0.5 L/min, and in the NF group, the gas flow rate was reduced to 2 L/min during the maintenance phase. In both groups, care was taken not to reduce the inspired oxygen fraction below 50%. In all patients, tidal volume was set at 6 mL/kg, and respiratory rate was adjusted to keep the EtCO_2_ concentration between 35 and 45 mmHg. When the surgeon informed the anesthesia team that 10 min were left to completion of the surgery, fresh gas flow increased back to 4 L/min with 100% oxygen support, and sevoflurane was discontinued. After seeing the patient could follow commands, extubation was performed, and transfer to the recovery unit was carried out.

### Monitorization and data collection

Age, sex, body mass index, ASA class, preoperative opioid consumption, duration of surgery, duration of general anesthesia, duration of PP, type of surgical procedure, and the total amount of consumed inhaled anesthetic were recorded for all study participants.

In the operating room, pulse rate, mean arterial pressure (MAP), peripheral hemoglobin oxygen saturation (spO_2_), and right and left sideregional cerebral oxygen saturation (Masimo’s O3 regional oximetry device) were measured before premedication and anesthesia induction, at 10 min after endotracheal intubation, at 10 min after PP, at 1^st^, 2^nd^, 3^rd^, 4^th^ and 5^th^ hours of operation, before repositioning to supine, after the resumption of supine position and at 5 min after extubation. End-tidal carbondioxide (EtCO_2_) was also monitored starting after intubation at the same time points asthe aforementioned measurements. NIRS was used to assess regional cerebral oxygenation (RCO) at the abovementioned time points. Regional cerebral oxygen saturation was assessed via the X Masimo (O3, Irvine, CA). Sensors of the device were placed on the right and left frontotemporal regions.

### Statistical analysis

The Shapiro–Wilk test and O-O plot were used to assess the normality. While continuous variables were presented as mean ± standard deviation or median (min-max) depending on the distribution of the variable, categorical variables were presented as numbers and %. Depend on the results of the normality test, the Mann–Whitney U test or independent samples t-test was used for the comparison of numeric variables between groups. Chi-squared test or Fisher’s exact test was performed for comparison of the categoric variables. To compare repeated measurements, the Friedman repeated measure test with post-hoc Durbin–Conover test was used. To prevent type I error, Bonferroni correction was used in posthoc comparisons.

All statistical analyses were performed using the SPSS 20.0 software package. (IBM Corp. Released 2012. IBM SPSS Statistics for Windows, Version 21.0. Armonk, NY: IBM Corp.). Graphs were performed with Excel. Statistically significance level was determined as p < 0.05.

As a result of the Power analysis using the G*Power program, the minimum number sample size (n) determined as 30 for each group for effect size d:0.725 and SD:2.7 with Power:0.80 and α:0.05.

## Results

A total of 46 patients (50% females) were randomized to either MF fresh gas (24 patients) or NF (22 patients) anesthesia. The mean age of the whole study group was 50.3 ± 15.1 years. Fourteen operations (30.4%) were performed by orthopedists, while 32 operations (69.6%) were carried out by neurosurgeons. All of the orthopedic procedures were posterior instrumentation. Neurosurgical procedures involved lumbar disk hernia surgery (18 patients), posterior stabilization (10 patients), posterior fossa tumor surgery (2 patients), and thoracal laminectomy (2 patients).

### Surgical and anesthetic features

ASA physical status classes were similar between MF and NF anesthesia groups. MF and NF anesthesia were regularly distributed over surgical departments and procedure types. Duration of anesthesia and surgery lasted significantly longer in the MF anesthesia group than in the NF anesthesia group. As a consequence, PP duration was also significantly longer in the former. The amount of anesthetic gas consumption was significantly lower in the MF anesthesia group compared with the NF anesthesia counterparts. Perioperative opioid medication requirements were comparable in both groups. Table 1 summarizes the surgical and anesthetic features of the MF and NF anesthesia groups.

**Table 1:** Comparison of minimal-flow and normal-flow anesthesia patients in terms of clinicodemographic characteristics, surgery, and anesthesia features.

	Characteristics	Low flow (n = 24)	Normal flow (n = 22)	P-value
Age (years)		50.8 ± 16.2	49.8 ± 14.2	0.822
Sex				
	Female	12 (50.0%)	14 (63.6%)	0.526
	Male	12 (50.0%)	8 (36.4%)	
	Body mass index (kg/m^2^)	25.9 ± 4.1	27.5 ± 4.7	0.210
ASA class				
	I	14 (58.3%)	12 (54.5%)	0.522
	II	10 (41.7%)	8 (36.4%)	
	III	0	2 (9.1%)	
	Amount of perioperative opioid used (mcg)	175 (0- 6000)	150 (50- 600)	0.399
	Duration of surgery (min)	222.5 (50- 610)	127.5 (80- 490)	**0.011**
	Duration of anesthesia (min)	267.5 (85- 675)	168.5 (125- 555)	**0.007**
	Duration of PP (min)	267.7 ± 146.2	184.4 ± 116.3	**0.037**
Surgical Department				
	Orthopedics and Traumatology	10 (41.7%)	4 (18.2%)	0.159
	Neurosurgery	14 (58.3%)	18 (81.8%)	
	Consumed anesthetic gas amount (mL)	41.9 ± 21.5	75.2 ± 47.3	**0.005**
Type of surgery				
	Posterior instrumentation	8 (33.3%)	4 (18.2%)	**0.152**
	Thoracal laminectomy	0	2 (9.1%)	
	Lumbar disc hernia repair	8 (33.3%)	8 (36.4%)	
	Posterior Stabilization	6 (25.0%)	4 (18.2%)	
	Posterior fossa tumor surgery	0	2 (9.1%)	
	Posterior instrument extraction	2 (8.3%)	0	
	Repeat lumbar disc hernia repair	0	2 (9.1%)	

### Monitorization parameters

#### Pulse rate

In both MF and NF groups, the mean pulse rate showed a decrease after PP. However, there was no difference between MF and high flow (HF) groups in this regard. After the resumption of supine posture, mean pulse rates increased slightly in both groups, but there was no difference between the groups again (Table 2).

**Table 2:** Comparison of pulse rate, mean arterial pressure, minimal alveolar concentration, and end-tidal carbon dioxide during measurement time points.

		Low flow (n = 24)	Normal flow (n = 22)	P-value
**Pulse rate** (beat/min)				
	Before anesthesia induction	80.1 ± 17.5	81.4 ± 9.1	0.755
	10 min after intubation	84.5 (53.0- 128.0)	95.0 (72.0- 120.0)	**0.025**
	10 min after PP	77.3 ± 18.9	84.5 ± 12.9	0.140
	1^st^ hour	71.0 ± 13.9	71.4 ± 7.3	0.911
	2^nd^ hour	73.5 ± 13.5	69.6 ± 7.7	0.267
	3^rd^ hour	72.3 ± 13.1	63.5 ± 4.0	**0.012**
	4^th^ hour	77.0 ± 17.3	64.7 ± 6.0	0.062
	Before repositioning to supine	73.5 (54.0- 110.0)	67.0 (59.0- 95.0)	0.509
	After supine repositioning	75.9 ± 20.7	71.7 ± 13.8	0.420
	5 min after extubation	77.7 ± 20.9	80.5 ± 11.1	0.565
**MAP** (mmHg)				
	Before anesthesia induction	102.6 ± 15.0	113.5 ± 15.3	**0.019**
	10 min after intubation	94.7 ± 20.1	104.8 ± 16.1	0.065
	10 min after PP	78.9 ± 15.6	88.8 ± 14.3	**0.029**
	1^st^ hour	79.5 (68.0- 106.0)	77.0 (55.0- 110.0)	0.202
	2^nd^ hour	87.9 ± 13.7	74.8 ± 9.8	**0.001**
	3^rd^ hour	83.0 (65.0- 114.0)	69.5 (57.0- 78.0)	**0.011**
	4^th^ hour	84.6 ± 11.9	73.3 ± 9.0	0.052
	Before repositioning to supine	86.9 ± 16.1	82.9 ± 19.0	0.442
	After supine repositioning	91.7 ± 14.4	80.4 ± 15.0	**0.013**
	5 min after extubation	93.8 ± 9.5	91.7 ± 17.1	0.627
**MAC**				
	Before anesthesia induction	---	---	
	10 min after intubation	0.7 (0.3-0.8)	0.7 (0.5-0.8)	0.107
	10 min after PP	0.8 (0.4-0.9)	0.8 (0.7-1.0)	0.876
	1^st^ hour	0.8 ± 0.1	0.7 ± 0.3	0.463
	2^nd^ hour	0.8 (0.1- 1.1)	0.9 (0.0- 1.0)	0.192
	3^rd^ hour	0.8 (0.0- 1.0)	0.8 (0.0- 1.0)	0.490
	4^th^ hour	0.8 (0.0- 1.0)	0.7 (0.0- 1.0)	0.562
	Before repositioning to supine	----		
	After supine repositioning	0.8 (0.0- 0.9)	0.8 (0.0- 1.0)	0.884
	5 min after extubation	0.3 (0.0- 0.9)	0.3 (0.0- 0.8)	0.537
**End-tidal carbon dioxide (EtCO2)** (mmHg)				
	Before anesthesia induction	----	----	
	10 min after intubation	35.0 (27.0- 39.0)	33.0 (28.0- 39.0)	0.250
	10 min after PP	32.3 ± 4.2	31.3 ± 2.9	0.316
	1^st^ hour	30.7 ± 3.2	29.7 ± 1.6	0.214
	2^nd^ hour	30.7 ± 3.1	28.5 ± 3.0	**0.032**
	3^rd^ hour	30.3 ± 2.9	27.5 ± 3.1	**0.047**
	4^th^ hour	28.2 ± 2.5	27.3 ± 2.3	0.491
	Before repositioning to supine	----		
	After supine repositioning	30.5 ± 2.7	30.0 ± 3.7	0.608
	5 min after extubation	31.6 ± 2.9	30.3 ± 4.0	0.213

#### Mean arterial pressure (MAP)

Baseline MAPs were different before anesthesia induction between the groups (102.6 ± 15.0 mmHg in the MF group and 113.5 ± 15.3 mmHg in the NF group, p = 0.019). After, PP blood pressure decreased significantly in both groups. However, MAP remained to be significantly higher in the NF group compared to the MF group. In the MF group, the mean MAP remained over 80 mmHg during the surgery. After the resumption of the supine position, MAP increased in both MF and NF groups. At this point, MAP was significantly higher in patients who were administered MF anesthesia. MAP values were comparable after extubation in both groups (Table 2).

#### End-tidal carbon dioxide (EtCO_2_) concentration

EtCO_2_ concentrations were comparable between the groups after intubation. With PP, in both groups, it showed a mild decrease. During the operation, at the 3^rd^ and 4^th^ hours, EtCO_2_ concentration was significantly higher in the MF anesthesia group. EtCO_2_ levels increased after repositioning to supine (Table 2).

#### Peripheral oxygen saturation (SpO_2_) and minimal alveolar concentration (MAC)

Peripheral oxygen saturation increased from 98% to 100% after PP in the MF group. Throughout the operation, including post-extubation status, spO_2_ did not show any reduction whatsoever (Table 3).

**Table 3:** Comparison of peripheral oxygen saturation, and right and left side regional cerebral oxygenation during measurement time points.

	**Low flow** (n = 24)	**Normal flow** (n = 22)	**P-value**
**Peripheral oxygen saturation (SpO2)** (%)			
Before anesthesia induction	96.6 ± 2.0	98.0 ± 1.6	**0.015**
10 min after intubation	98.0 (97.0-100)	98.0 (97.0-100)	0.296
10 min after PP	100 (97.0-100)	98.0 (96.0-100)	**0.031**
1^st^ hour	98.5 (98-98)	97 (97-97)	0.286
2^nd^ hour	99.5 (99-100)	99 (99-99)	0.089
3^rd^ hour	99 (99-99)	100 (100- 100)	0.237
4^th^ hour	99.5 (99-100)	100 (100- 100)	0.562
Before repositioning to supine	100 (100- 100)	100 (100- 100)	>0.999
After supine repositioning	100 (98- 100)	100 (97- 100)	0.160
5 min after extubation	100 (98- 100)	100 (98- 100)	0.375
**RCO right side** (%)			
Before anesthesia induction	70.4 ± 9.9	65.7 ± 5.0	**0.046**
10 min after intubation	70.9 ± 9.6	69.5 ± 6.6	0.547
10 min after PP	68.2 ± 8.3	67.1 ± 6.3	0.608
1^st^ hour	66.0 ± 7.6	65.3 ± 4.5	0.693
2^nd^ hour	64.5 ± 7.6	62.1 ± 5.6	0.255
3^rd^ hour	63.5 ± 6.7	57.1 ± 6.3	**0.028**
4^th^ hour	61.8 ± 6.0	58.5 ± 7.3	0.371
Before repositioning to supine	61 (60- 74)	67 (67-67)	0.643
After supine repositioning	65.6 ± 6.9	67.6 ± 6.0	0.297
5 min after extubation	71.8 ± 11.1	69.9 ± 7.4	0.500
**RCO left side** (%)			
Before anesthesia induction	71.6 ± 9.2	63.9 ± 6.1	**0.002**
10 min after intubation	71.0 ± 8.6	64.5 ± 8.1	**0.012**
10 min after PP	71.0 ± 9.0	65.2 ± 7.3	0.021
1st hour	68.7 ± 7.6	63.8 ± 6.1	**0.019**
2nd hour	67.8 ± 8.5	60.7 ± 7.8	**0.012**
3rd hour	67.2 ± 8.2	57.4 ± 6.6	**0.005**
4th hour	65.3 ± 6.3	56.3 ± 8.2	**0.045**
Before repositioning to supine	68.7 ± 4.7	68.5 ± 0.7	0.936
After supine repositioning	68.4 ± 7.9	63.6 ± 8.1	**0.049**
5 min after extubation	72.4 ± 8.3	66.3 ± 7.7	**0.013**

#### Regional cerebral oxygenation (measured by NIRS)

**Figure 2:**
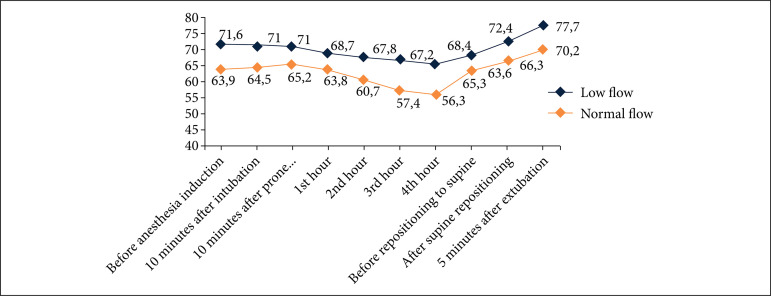
Left-sided cerebral oxygen saturation measurements during study time-points.

**Figure 3:**
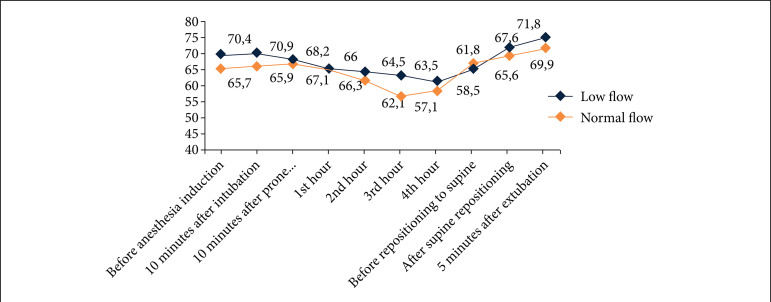
Right-sided cerebral oxygen saturation measurements during study time-points.

At baseline evaluation before anesthesia induction, RCO was significantly higher both at the right and left sides in the MF group compared to the NF group. This difference continued throughout the operation on the left side. However, this difference disappeared 10 min after intubation on the right side. Placing the patients in a prone position did not cause any alteration in RCO on the right side in any of the groups. However, on the left side, mean RCO decreased after PP in both MF and NF groups (Fig. 2). Supine repositioning did not change RCO in any of the groups on the right side. However, it decreased RCO on the right side in the MF group after supine repositioning; and this decrease was not statistically significant (Table 3, Fig. 3).

## Discussion

The purpose of the study to evaluate the impact of MF inhaled anesthesia coupled with PP on cerebral oxygenation and hemodynamic parameters. The major findings of the present study can be summarized as follows: 1) Anesthetic gas consumption was significantly lower in the MF anesthesia group; 2) PP decreased pulse rate irrespective of the flow type of anesthesia. However, pulse rates were mostly comparable between MF and NF anesthesia groups; 3) Although significantly lower before anesthesia induction in the MF group, pulse rate decreased in both MF and NF groups after PP and usually remained significantly higher in the MF group compared to the NF group throughout the rest of the procedures; 4) Left-sided regional cerebral oxygen saturations were consistently and significantly higher in the MF group compared to the NF group. PP decreased RCO on the right side, but not on the left side, in both anesthesia groups; 5) There was no discernable effect of anesthesia flow or PP on peripheral oxygen saturation in any of the groups; 6) Anesthesia depth (assessed by MAC) was comparable between the MF and NF groups at all measurement points during anesthesia induction and surgery.

Surgical procedures dealing with the spine usually require PP of the patients. This unfamiliar position might lead to some alteration in the respiratory and hemodynamic functioning of the body. First, when the thorax and abdomen are positioned in the prone position, intraabdominal and intrathoracic pressures increase with a resultant reduction in venous return to the heart. Arterial blood pressure and cardiac output decrease. Increased intrathoracic pressure also leads to a secondary increase in intracranial pressure through increased central venous pressure^12^^,^ 13.

Several studies sought to find answers to whether these hemodynamic changes can lead to clişnically important hemodynamic and oxygenation alterations in the cerebral circulation. Bombardieri et al. performed a study in which they investigated the impact of PP on cerebral perfusion in patients undergoing posterior lumbar surgery. The authors evaluated cerebral blood flow velocity with transcranial Doppler ultrasonography. They found an insignificant increase in mean arterial blood pressure after PP. Cerebral perfusion was not affected significantly by PP , either^14^. In a prospective observational study, Babakhani and colleagues assessed cerebral oxygenation in patients undergoing lumbar spine surgery in the prone position. The authors used NIRS to evaluate RCO as in this study. The results of the study showed that after 30 and 60 min of PP, cerebral oxygenation significantly and bilaterally decreased compared to the values measured at the supine position. At 90 min, the cerebral oxygenation values returned to baseline values. Moreover, oxygen desaturation co-occurred with significant blood pressure and pulse rate decreases^9^. In contrast to the findings of the latter study, another prospective observational study found a significant increase in cerebral oxygen saturation assessed by the NIRS method in patients undergoing orthopedic surgery in the prone position^10^. The results showed a decrease with PP only on the right side of the brain. The decrease was present in both flow groups but was not statistically significant. Peripheral oxygen saturation did not change with PP . Similar to the results of Babakhani et al., our results showed decreases in both MAP and pulse rate in both flow groups with PP. These changes did not reach statistical significance and were more or less maintained during the surgical procedure thereafter. In sum, with PP, right-sided RCO, MAP, and pulse rate decreased nonsignificantly. These changes were also nonconsequential clinically.

Despite several enticing advantages mentioned in the introduction section, MF anesthesia has several concerns, as well. Two of them are inadequate anesthesia depth and hypoxia. Some studies evaluated LF anesthesia in these regards in various surgical settings. Kupisiak et al.^7^, in a randomized controlled trial, evaluated the impact of LF anesthesia on anesthesia depth and cerebral oxygenation in patients undergoing laparoscopic cholecystectomy. The authors reported nonsignificant and comparable changes in hemodynamics and cerebral oxygenation in LF and HF anesthesia. In both groups, anesthesia depth assessed by the bispectral index was adequate^7^. Another randomized controlled study compared LF (0.75 L/min) and NF (1.5 L/min) anesthesia on cerebral oxygenation and anesthesia depth in morbidly obese patients undergoing bariatric surgery^6^. The study results demonstrated comparable and clinically insignificant changes both in RCO and anesthesia depth. Both groups showed similar values of cerebral oxygen saturation and bispectral index. Another randomized study involving patients undergoing septorhinoplasty compared MF and HF anesthesia in terms of cerebral perfusion assessed by the NIRS method. Although controlled hypotension was utilized in these operations, the study did not report any difference between the flow groups regarding RCO^11^. To the best of our knowledge, no study has investigated yet whether the combination of prone position with MF anesthesia can lead to systemic hemodynamic and cerebral perfusion changes. Our results showed some hemodynamic changes between MF and NF groups. Despite higher MAP levels before anesthesia induction, starting from PP, MAP values were significantly higher in the MF group. Pulse rates tended to be lower in the NF group during the surgery, but there was no significant difference between the groups. Similarly, peripheral oxygen saturation and EtCO_2_ concentration values were similar (except for the 2^nd^ and 3^rd^ hours for EtCO_2_, which was significantly higher in the NF group). Left-sided NIRS measured RCO was significantly better in the MF group throughout the surgery. However, these values were comparable on the right side. In sum, MF anesthesia was associated with higher MAP and higher left-sided cerebral oxygen saturation values compared to NF anesthesia patients. However, apart from left-sided 3^rd^ and 4^th^-hour cerebral saturations in the NF group, all values of NIRS cerebral saturation were within normal levels (60–75%).

Some limitations of the current study are worthy of mention. First, the bispectral index was not included to evaluate anesthesia depth. Instead, minimum alveolar concentration has been used. Second, some studies follow patients after general anesthesia and evaluate mental state before and after the operation to unravel the effects of alterations of cerebral oxygenation on cognitive functions. Cognitive functions were not evaluated in the study. However, no cerebral oxygen desaturation was observed in the MF group compared to the NF group. Lastly, the right and left regional cerebral oxygen saturation values at baseline were significantly different between the LF and NF groups, significantly higher in the lef hand side. Thus, this might have led to the discrepant results we attained.

## Conclusion

For the first time in the literature, this study evaluated the impact of PP along with MF anesthesia on systemic hemodynamics and RCO. The results of the present study revealed that MF anesthesia in patients undergoing surgery in the prone position did not cause a reduction in cerebral oxygenation compared to NF. PP also caused clinically nonsignificant changes in hemodynamic parameters. Anesthesia depth was acceptable in the MF anesthesia group as well. MF anesthesia in the prone position was safe in terms of systemic hemodynamics and cerebral oxygenation.

## Data Availability

The data will be available upon request;
